# Secondary Traumatic Stress and Burnout in Healthcare Workers during COVID-19 Outbreak

**DOI:** 10.3390/ijerph18010337

**Published:** 2021-01-05

**Authors:** Graziella Orrù, Francesca Marzetti, Ciro Conversano, Guido Vagheggini, Mario Miccoli, Rebecca Ciacchini, Eugenia Panait, Angelo Gemignani

**Affiliations:** 1 Department of Surgical, Medical and Molecular Pathology, Critical and Care Medicine, University of Pisa, 56121 Pisa, Italy; graziella.orru@unipi.it (G.O.); dr.francescamarzetti@gmail.com (F.M.); ciro.conversano@unipi.it (C.C.); rebecca.ciacchini@gmail.com (R.C.); angelo.gemignani@unipi.it (A.G.); 2 Weaning and Cardio-Pulmonary Rehabilitation Unit, Auxilium Vitae Rehabilitation Centre, 56148 Volterra, Italy; 3 Fondazione Volterra Ricerche ONLUS, 56148 Volterra, Italy; eugeniapanait57@gmail.com; 4 Department of Clinical and Experimental Medicine, University of Pisa, 56121 Pisa, Italy; mario.miccoli@unipi.it

**Keywords:** COVID-19, secondary traumatic stress, burnout, health care workers

## Abstract

(1) Background: The present study aims to assess the level of professional burnout and secondary traumatic stress (STS), and to identify potential risk or protective factors among health care workers (HCWs) during the coronavirus disease 2019 (COVID-19) outbreak.; (2) Methods: This cross-sectional study, based on an online survey, collected demographic data and mental distress outcomes from 184 HCWs from 1 May 2020, to 15 June 2020, from 45 different countries. The degree of STS, perceived stress and burnout was assessed using the Secondary Traumatic Stress Scale (STSS), the Perceived Stress Scale (PSS) and Maslach Burnout Inventory Human Service Survey (MBI-HSS) respectively. Stepwise multiple regression analysis was performed to identify potential risk and protective factors for STS; (3) Results: 184 HCWs (M = 90; Age mean: 46.45; SD: 11.02) completed the survey. A considerable proportion of HCWs had symptoms of STS (41.3%), emotional exhaustion (56.0%), and depersonalization (48.9%). The prevalence of STS was 47.5% in frontline HCWs while in HCWs working in other units it was 30.3% (*p* < 0.023); 67.1% for the HCWs exposed to patients’ death and 32.9% for those HCWs which were not exposed to the same condition (*p* < 0.001). In stepwise multiple regression analysis, perceived stress, emotional exhaustion, and exposure to patients’ death remained as significant predictors in the final model for STS (adjusted R2 = 0.537, *p* < 0.001); (4) Conclusions: During the current COVID-19 pandemic, HCWs facing patients’ physical pain, psychological suffering, and death are more likely to develop STS.

## 1. Introduction

The health emergency due to the COVID-19 outbreak has heavily impacted the psychological and emotional wellbeing of the general population [1,2] and healthcare workers (HCWs). In the frontline HCWs, different sources of psychological distress have been reported, such as uncertainty of the disease progression (short- and long-term effects), treatment, lack of personal protective equipment (PPE), physical exhaustion, overwhelming workload, concerns about the direct exposure to COVID-19 at work. In particular, the latter is associated with the fear of getting infected or spreading the infection among colleagues and families members [3,4,5,6,7,8]. Additionally, frontline HCWs took care of patients who were both physically and psychologically suffering from the emergency (vicarious traumatization), and, as a consequence, they were exposed to the risk of developing secondary traumatic stress disorder [9,10].

Results emerging from empirical researches, carried out in comparable periods such as the Severe Acute Respiratory Syndrome (SARS) or the Middle East Respiratory Syndrome (MERS) outbreaks, highlighted that HCWs experienced high levels of stress, anxiety and depressive symptoms [11,12], psychological distress [13] and post-traumatic stress symptoms that include avoidance, hyperarousal and insomnia [11,14,15]. As expected, frontline HCWs experienced greater psychological distress compared to HCWs with secondary roles [11].

According to recent studies, Chinese HCWs directly caring for COVID-19 patients showed higher levels of distress, anxiety and insomnia while compared to HCWs involved in secondary roles [16,17,18,19]. Medical HCWs showed a higher prevalence of insomnia, depressive symptoms, anxiety and obsessive-compulsive symptoms compared to nonmedical healthcare workers [18]. Two recent systematic reviews and meta-analysis underlined the higher prevalence of depression, anxiety, and insomnia among HCWs during the COVID-19 outbreak [20,21].

Direct exposure to the high level of distress during the COVID-19 pandemic, seems to increase the risk of long term consequences such as post-traumatic stress, depressive symptoms [22] or professional burnout with adverse outcomes for the whole organization [23]. Professional burnout or occupational burnout has been defined by Maslach [24] as a *“response to prolonged and chronic stress at the workplace, characterized by three dimensions: emotional exhaustion, depersonalization and reduction of personal abilities”.* This condition appears to be predominant in the medical health care professionals rather than in others [25,26]. Schanafelt et al. [27] reported that the overall mean rate of physician burnout rose from 45.5% in 2011 to 54.4% in 2014 (*p* < 0.001). It is characterized by a gradual development over time accompanied by reduced professional satisfaction which can lead to a poorer ability to judge, late or inadequate responses to changes in the clinical context, and lack of patient confidence in HCWs, compromising professional performance [26,28,29,30,31]. 

Another consequence of the COVID-19 outbreak may be represented by pathologic secondary traumatic stress (STS). Figley [32] defined STS as *“the stress deriving from helping others who are suffering or who have been traumatized”*. Some authors use the terms STS or compassion fatigue or vicarious traumatization interchangeably [33]. In ordinary situations, because of the implications of their professional sector, HCWs may be at higher risk of developing pathological secondary traumatization and this is particularly true now more than usual, considering the present emergency. Protective factors such as resilience, self-efficacy and perceived social support may be able to reduce STS and anxiety symptoms [34]. According to Bellolio [35], burnout arises gradually and progressively, both in professional and/or personal life, as a result of unsuccessful coping strategies, while STS is an acute stress reaction, secondary to the relationship with traumatized patients.

Interventions aimed at healthcare professionals should, through the support of psychologists, focusing on the management and containment of maladaptive behaviours, emotional disorders and distress [17,36,37,38]. Even if different kinds of psychological interventions have been available, results remain unclear and HCWs often refuse to participate [17,39,40]. 

The present study aims to assess the psychological distress in terms of perceived stress, professional burnout and STS, and to identify potential risk or protective factors, among HCWs during the COVID-19 outbreak all over the world. The sample of HCWs was evaluated when confinement measures were in place (i.e., lockdown or partial limitations).

## 2. Materials and Methods 

### 2.1. Study Design and Participants

A cross-sectional international survey addressed to HCWs was conducted from 1 May to 15 June 2020. The Ethics Committee of the University of Pisa approved our study survey and procedures of informed consent before the formal survey.

A link to the above-mentioned survey was sent directly to HCWs and the European Respiratory Society (ERS) and was published at the following link: https://www.ersnet.org/research/covid-19-surveys. The informed consent was available online and was provided to all participants before enrolment. The survey was anonymous, and confidentiality of information was assured. 

One-hundred and eighty-four HCWs from 45 Countries and 5 continents completed the online survey. Participants were eligible if the following criteria were met: 1. working in health care during COVID-19 outbreak; 2. gave informed consent. 

### 2.2. Materials

Socio-demographic data were self-reported by participants and included gender, age, Country, education, occupation, seniority, civil status, number of children and pathologies. 

Data related to personal and professional experience during the COVID-19 outbreak were also included: actual lockdown policies of the home Country, COVID-19 test status (where known, and positive), the severity of symptoms of family members or friends infected by COVID-19, direct involvement in the assistance of COVID-19 patients, daily work with COVID-19 patients, exposure to patients’ death. Respondents were also asked to evaluate how the emergency was managed by the organization/hospital (1 very bad management—10 very good management) and the perceived degree of emergency (1 COVID-19 is not a real emergency—10 it is a real emergency). 

Perceived Stress Scale (PSS): The PSS is a 10-item questionnaire designed to assess the degree to which external demands seem to exceed the individual’s perceived ability to cope [41]. Respondents are asked to indicate how frequently they felt or thought the certain way in the last month on a 5-point Likert scale ranging from 0 (never) to 4 (very often). The PSS total score is calculated by summing up the item scores, with a higher score indicating higher perceived stress. Score range from 0 to 40. 

Secondary Traumatic Stress Scale (STSS): The STSS is a 17-item questionnaire designed to measure the negative impact of indirect exposure to traumatic events in HCWs caring for suffering or traumatized clients. The traumatic stressor for HCWs is identified as exposure to patients. Respondents are asked to indicate how frequently the item was true for them in the past seven days on a 5-point Likert scale ranging from 1 (never) to 5 (very often). The STSS has a global score and three subscales: Intrusion (five items), that refers to recurrent and intrusive distressing recollections of patients, including images, thoughts, or perceptions; avoidance (seven items), that measures the avoidance of stimuli associated with the care of patients and the numbing of general responsiveness; arousal (five items), that assess symptoms such as irritability, hypervigilance, difficulty concentrating. The STSS global score is calculated by summing up all the item scores, with a higher score indicating a higher frequency of symptoms. The total score ranges from 17 to 85 and is categorized into no/little (17–28), mild (28–37), moderate (38–43), high (44–48), and severe (49–85) levels of secondary trauma [42]. 

Maslach Burnout Inventory Human Service Survey (MBI-HSS): The MBI-HSS is a 22-item questionnaire that assesses professional burnout among people involved in the care and social services [24]. Respondents are asked to indicate the frequency with which they experience certain feelings or attitudes on a 7-point Likert scale ranging from 0 (never) to 6 (every day). MBI-HSS is composed by three subscales: Emotional Exhaustion (MBI-EE, 9 items), that assess the feelings of being emotionally overextended by one’s work; Depersonalization (MBI-D, 5 items), that measures unfeeling and impersonal response to care; Personal Accomplishment (MBI-PA, 8 items), that assess the feelings of competence, the perceived effectiveness on the job. The scores for each subscale are not combined into a global score: they are separated, with different cut-off points (MBI-EE: low 0–16, moderate 17–26, high 27–54; MBI-D: low 0–6, moderate 7–12, high 13–35; MBI-PA: low 0–31, moderate 32–38, high 39–48). 

14-Item Resilience Scale (RS-14): The RS-14 is a 14-item questionnaire to assess the individual ability to withstand or adaptively recover from stress [43]. Items are evaluated on a 7-point Likert scale ranging from 1 (strongly disagree) to 7 (strongly agree). Total score ranges from 14 to 98, with higher scores indicating greater resilience. RS-14 yields reliable scores, coefficient alphas of 0.90 and greater [43]. 

General Self-Efficacy Scale (GSE): The GSE is a 10-item instrument that measures the perceived self-efficacy, the belief that one can successfully cope with an adverse situation or stressor [44]. GSE explicitly refers to personal agency, that is, the belief that one’s own actions are responsible for successful outcomes. Each item is evaluated on a 4-point Likert scale, scored from 1 (not at all true) to 4 (completely true). The total score is calculated by finding the sum of all items and ranges between 10 and 40, with a higher score indicating higher self-efficacy. 

### 2.3. Statistical Analysis

Data are presented as mean and standard deviation (SD). Comparisons between groups were performed using *t*-test for independent samples and chi-squared test for categorical variables. Pearson’s correlation was computed to evaluate the relationship between psychological distress variables, protective factors, socio-demographic characteristics, and COVID-19 experience. The statistically significant variables were selected for the multivariate model and a second selection was carried out with stepwise procedure. Stepwise multiple linear regression analysis was performed to determine potential risk and protective factors for secondary traumatic stress. *p* values < 0.05 were considered statistically significant. The analysis was conducted using SPSS statistics version 21 (IBM, Armonk, NY, USA).

## 3. Results

### 3.1. Demographic Characteristics and Outcomes of Psychological Distress in Total Cohort

A total of 184 HCWs (M = 90; mean age: 46.45; SD: 11.02) completed the survey questionnaire and the professions were the following: physicians (n = 138; 75.0%), nurses (n = 10; 5.4%), surgeons (n = 3; 1.6%), psychologists (n = 2; 1.1%) and other health professionals (n = 31; 16.8%). The demographic characteristics of the sample are summarized in Table 1.

118 HCWs (64.1%) were frontline and directly involved in the care of COVID-19 patients while 66 HCWs (35.9%) were involved in different units. Ten out of 184 HCWs (5.6%) were infected by COVID-19 and 57 HCWs (31.0%) had one or more family members infected by COVID-19. The mean of respondents was mainly satisfied with how the organization managed the critical situation (mean = 7.73, SD = 1.75) and did not perceive COVID-19 outbreak as a severe emergency (mean = 4.28, SD = 3.15) (Table 2). The sample of HCWs was evaluated when the confinement measures were in place. Specifically, 98 HCWs (53.3%) were evaluated when their countries were in a strict lockdown and the remaining 86 HCWs (47.7%) were assessed when partial limitations were established.

A considerable proportion of HCWs had symptoms of secondary traumatic stress (STSS ≥ 38, moderate to severe symptoms, 41.3%), emotional exhaustion (MBI-EE ≥ 17, moderate to high, 56.0%), and depersonalization (MBI-D ≥ 7, moderate to high, 48.9%). The mean (SD) scores on the PSS, STSS and subscales, MBI-HSS subscales, GSE, and RS-14 are shown in Table 3.

Correlation analysis between psychological distress, secondary traumatic stress, professional burnout, protective factors, demographics, and professional experience during the COVID-19 outbreak is reported in Table 4.

### 3.2. Demographic Characteristics and Outcomes of Psychological Distress: Differences between Subgroups

Female HCWs showed significantly higher scores than male HCWs on STSS Intrusion subscale (*p* = 0.013), and on MBI-EE (*p* = 0.007). HCWs without children exhibited significantly higher scores on global STSS (*p* < 0.001) and all subscales (Intrusion, *p* = 0.003; Avoidance, *p* < 0.001; Arousal, *p* = 0.001), PSS (*p* = 0.001), MBI-EE (*p* = 0.002) and MBI-D (*p* = 0.033), compared to the colleagues with one or more children, and lower GSE (*p* = 0.031). HCWs with family members or friends infected by COVID-19 displayed significantly higher scores on PSS (*p* = 0.013), Intrusion (*p* = 0.028) and Arousal subscale (*p* = 0.057) (Table 5).

The comparison between HCWs working in Countries with hard lock-down policies (HLD, n = 96) and HCWs working in Countries with softer lock-down policies (SLD, n = 80) showed that HCWs working in SLD conditions exhibited lower MBI-D (*p* = 0.054) scores and higher MBI-PA (*p* = 0.019) and RS-14 (*p* = 0.005) scores.

#### 3.2.1. Frontline HWCs 

The prevalence of STS (STSS ≥ 38, moderate to severe symptoms) in frontline HCWs (F-HCWs, n = 118) was 47.5% while a lower rate (30.3%) was detected for the HCWs working in other units (OU-HCWs, n = 66) (*p* < 0.029). F-HCWs exhibited significantly higher scores on STSS Intrusion subscale (*p* = 0.016) than OU-HCWs, but significantly lower scores on MBI-D (*p* = 0.029). 

Correlation analysis in F-HCWs subgroup showed that the higher Intrusion scores were significantly and positively associated to PSS (*p* < 0.001), MBI-EE (*p* < 0.001), MBI-D (*p* < 0.001), female gender (*p* = 0.004), hours per day spent with patients (*p* = 0.020) and exposure to patients’ deaths (*p* = 0.001). Meanwhile, they were negatively related to age (*p* = 0.003), the number of children (*p* = 0.002), GSE (*p* = 0.002), RS-14 (*p* = 0.036). In OU-HCWs subgroup, a positive significant correlation was found between Intrusion and PSS (*p* < 0.001), MBI-EE (*p* < 0.001), MBI-D (*p* < 0.001), while a negative relation was found with RS-14 (*p* = 0.003) and the number of children (*p* = 0.021) (Table 6).

The lower MBI-D scores found in F-HCWs had a significant and positive association with PSS (*p* < 0.001), STSS (*p* < 0.001), MBI-EE (*p* < 0.001) and the number of hours per day spent with patients (*p* = 0.033) and a significative negative association with age (*p* = 0.033), GSE (*p* < 0.001), RS-14 (*p* = 0.003), and MBI-PA (*p* = 0.006). In OU-HCWs a positive significant correlation was found between MBI-D and PSS (*p* = 0.003), STSS (*p* < 0.001), MBI-E (*p* < 0.001) meanwhile a negative correlation was reported with RS-14 (*p* < 0.001).

#### 3.2.2. Exposure to Patients’ Death as a Risk Factor for Secondary Traumatic Stress

The prevalence of secondary traumatic stress (STSS ≥ 38, moderate to severe symptoms) in HCWs exposed to infected patients’ death (E-HCWs, n = 94) was 54.3% while it was 27.8% in HCWs who were not exposed (NE-HCWs, n = 90) (*p* < 0.001). E-HCWs also reported significantly higher scores on PSS (*p* = 0.031), STSS (*p* < 0.001), and all subscales (Intrusion, *p* < 0.001; Avoidance, *p* = 0.002; Arousal, *p* = 0.001) than NE-HCWs. 

Stepwise multiple linear regression analysis was performed to find out the predictors of STSS in the total cohort. In the final model for STSS, exposure to patients’ deaths, PSS, and MBI-EE scores remained as significant predictors, with a good level of fit with the data (adjusted R2 = 0.537). Significant protective factors, such as resilience or self-efficacy, were not found. Concerning F-HCWs and OU-HCWs subgroups, stepwise multiple regression analysis was performed to identify the predictors of Intrusion symptoms. In F-HCWs the final model for Intrusion had PSS, MBI-EE, MBI-D, female gender, and exposure to patients’ deaths as significant predictors, with a good level of fit with the data (adjusted R2 = 0.486). Meanwhile, in the final regression model for Intrusion in OU-HCWs (adjusted R2 = 0.306), only PSS and MBI-D remained as significant predictors. The results are summarized in Table 7.

## 4. Discussion

The present study aimed to assess psychological distress, secondary traumatic stress and professional burnout in HCWs during the COVID-19 outbreak. A prevalence of secondary traumatization among HCWs ranging from 4% to 13% was described in studies before the COVID-19 outbreak [9], and during the COVID-19 outbreak a considerable proportion of HCWs experienced mood and sleep disturbances [20,21]. Our findings showed symptoms of moderate to severe secondary traumatization in a higher proportion of the total respondents, exceeding 40%. In particular, women exhibited a greater effect than men (47.3% vs. 34.4%) and this is line with previous studies [45]. Therefore, the respective HCWs’ situation appears to be critical, with a prevalence even higher in frontline HCWs (47.5%) and in HCWs exposed to infected patients’ death (67.1%). 

Secondary traumatic stress was positively associated with: (i) the amount of time spent with COVID-19 patients; (ii) a great exposure to COVID-19 patients’ deaths; (iii) the severity of symptoms of family members or friends infected by COVID-19. A significant regression model was obtained, and STS was positively predicted by perceived stress, emotional exhaustion, and exposure to patients’ death, confirming the central role that failed care-taking efforts have in the development of secondary traumatization. In frontline HCWs, the relationship between STS, specifically intrusion symptoms, and exposure to patients’ death as predictors was confirmed, meanwhile, it was not observed in HCWs working in other units. No significant protective factors were found. Considering these findings, we reasonably hypothesize that the observed high level of STS is consistent with the actual outbreak and therefore its potential long-term consequences should be considered. 

The prevalence of professional burnout is similar to previous findings and is over 50% [26,27]. No significant differences in frontline HCWs or HCWs exposed to patients’ death were found for the prevalence of professional burnout, suggesting that it is not so closely related to the COVID-19 outbreak. Even if in our study professional burnout correlates with secondary traumatization, that may be due to the partial overlapping of constructs [9]. Bellolio et al. [35] underlined that burnout is a result of the mismatch between the nature of the job and the nature of the person who does the job, it’s gradual and it arises from daily life, through continuous negative experiences, without a necessarily traumatic character [26,35,46]. Secondary traumatic stress is instead an acute reaction, secondary to a relationship, that arises when rescue care-taking efforts are unsuccessful [35]. Our findings appear to be in accordance with this distinction. 

In our study, frontline HCWs had significant higher scores on secondary traumatic stress, intrusion subscale, meanwhile, they exhibited a significantly lower score on burnout, depersonalization subscale, when compared to HCWs involved in other units. This may be since intrusion symptoms are characteristics of traumatic stress reactions, that particularly emerged during the COVID-19 outbreak in frontline HCWs and may be distinctive from other symptomatology such as professional burnout. Further investigations are required. It is also possible that, in our respondents, high levels of burnout and perceived stress were already present before the COVID-19 outbreak, as similar levels were reported in HCWs in previous studies [26,27]. This point needs to be clarified with longitudinal studies. 

The present study suffers from a number of limitations and weaknesses. The complexity of the survey and the time required to fill the questionnaires limited the number of participants, moreover in a period in which the workload was overwhelming and the main part of respondents was directly involved with patients. The limited number of participants make it difficult to generalize the results to the whole HCWs population. The cross-sectional nature of the study and the lack of longitudinal follow-up do not allow inferences about the causal relations among the variables, and the long-term consequences of the psychological outcomes found. Additionally, we did not evaluate possible differences between the healthcare systems, their specific organizational policies or HCWs workload. Another major challenge encountered was the heterogeneity of the confinement measures in place in different countries. The above-mentioned issues would have a significant impact both on the STS and burnout. Therefore, these limitations preclude robust conclusions.

Further prospective studies could also clarify the relation between burnout, secondary traumatic stress, and protective factors. Long-term implications for HCWs mental health and consequences of personal and organizational factors are worth further investigation.

## 5. Conclusions

Our findings suggest that the COVID-19 outbreak had an impact on the more frequent direct exposure to the patients’ physical pain, psychological suffering, and death, which increased secondary traumatization in HCWs. Further investigations are required, to better clarify the longitudinal course of the effects of traumatization and the occurrence of long-term pathologic consequences. The prevalence of STS symptoms in HCWs and the long-term changes in their mental wellbeing need to be further investigated with longitudinal studies. Large scale screening in highly exposed or more vulnerable HCWs are needed, to identify subjects requiring targeted treatment and prevent long term psychological and health consequences.

## Figures and Tables

**Table 1:** Demographic characteristics.

n = 184		Mean (SD)—Range	n (%)
Age		46.45 (11.02)—24–74	
Gender	F		93 (50.5%)
	M		90 (48.9%)
	Prefer not to say		1 (0.6%)
Education	Bachelor’s Degree		20 (10.9%)
	Bachelor’s Degree + Specialization		61 (33.2%)
	Master		35 (19.0%)
	PhD		68 (37.0%)
Occupation	Physician		138 (75.0%)
	Surgeon		3 (1.6%)
	Nurse		10 (5.4%)
	Psychologist		2 (1.1%)
	Other health professionals		31 (16.8%)
Seniority		19.90 (11.58)—0–50	
Civil status	Single		31 (16.8%)
	Married/with partner		142 (77.2%)
	Divorced		11 (6.0%)
Children	None		53 (28.8%)
	One		34 (18.5%)
	Two		70 (38.0%)
	Three or more		27 (14.7%)
Pathologies	None		129 (70.1%)
	Cardiovascular pathology		10 (5.4%)
	Psychiatric condition		1 (0.5%)
	Chronic pain		7 (3.8%)
	Chronic respiratory conditions		18 (9.8%)
	Diabetes		6 (3.3%)
	Other		13 (7.1%)

**Table 2:** COVID-19 outbreak individual experience.

n = 184		Mean (SD)—Range	n (%)
Lock-down	Completely		10 (5.4%)
	Phase 2		97 (52.7%)
	Phase 3		51 (27.7%)
	All open		26 (14.1%)
Care of COVID-19 patients	No, involved in other units		66 (35.9%)
	Yes, frontline		118 (64.1%)
Hour per day with COVID-19 patients	0		65 (35.3%)
	1 to 4 h per day		56 (30.4%)
	4 to 8 h per day		40 (21.7%)
	More than 8 h per day		23 (12.5%)
Exposure to COVID-19 patients’ death	No		90 (48.9%)
	Yes, sometimes		64 (34.8%)
	Yes, very often		30 (16.3%)
Positivity to COVID-19	No		174 (94.6%)
	Yes		10 (5.6%)
Family or friends infected by COVID-19	No		127 (69.0%)
	Yes, without complications		26 (14.1%)
	Yes, with hospitalization		27 (14.7%)
	Yes, and one or more died		4 (2.2%)
Management of the critical situation		7.73 (1.75)—1–10	
COVID-19 is a severe emergency		4.28 (3.15)—1.10	

**Table 3:** Questionnaire scales and subscales scores and prevalence in total cohort.

n = 184	Mean (SD)	n (%)
PSS	16.80 (6.27)	
STSS	36.41 (12.79)	
No STSS		65 (35.4%)
Mild STSS		43 (23.4%)
Moderate STSS		26 (14.1%)
High STSS		8 (4.3%)
Severe STSS		42 (22.8%)
Intrusion	10.45 (4.08)	
Avoidance	14.50 (5.32)	
Arousal	11.46 (4.50)	
MBI-HSS		
EE	19.66 (11.12)	
Low		81 (44.0%)
Moderate		56 (30.5%)
High		47 (25.5%)
D	7.53 (5.51)	
Low		94 (51.1%)
Moderate		58 (31.5%)
High		32 (17.4%)
PA	32.48 (8.16)	
Low		70 (38.0%)
Moderate		70 (38.0%)
High		44 (24.0%)
GSE	30.75 (5.73)	
RS-14	78.61 (12.71)	

Abbreviations: PSS = Perceived Stress Scale; STSS = Secondary Traumatic Stress Scale; MBI-HSS = Maslach Burnout Inventory Health Services Scale; EE = Emotional Exhaustion; D = Depersonalization; PA = Personal Accomplishment; GSE = General Self-Efficacy Scale; RS-14 = 14-item Resilience Scale.

**Table 4:** Significant Pearson correlation coefficients r: association between perceived stress, secondary traumatic stress, professional burnout, protective factors, demographics, and COVID-19 individual experience in the total cohort (n = 184).

	1	2	3	4	5	6	7	8	9	10	11	12	13	14	15	16
1.PSS	-															
2.STSS_	0.633 **	-														
3.Intrusion	0.540 **	0.893 **	-													
4.Avoidance	0.591 **	0.939 **	0.752 **	-												
5.Arousal	0.611 **	0.923 **	0.742 **	0.805 **	-											
6.MBI-EE	0.555 **	0.645 **	0.562 **	0.596 **	0.621 **	-										
7.MBI-D	0.416 **	0.480 **	0.443 **	0.472 **	0.404 **	0.586 **	-									
8.MBI-PA	−0.307 **	−0.034	−0.018	−0.090	−0.006	−0.095	−0.192 **	-								
9.GSE	−0.381 **	−0.244 **	−0.214 **	−0.268 **	−0.182 *	−0.259 **	−0.190 **	0.466 **	-							
10.RS-14	−0.337 **	−0.293 **	−0.234 **	−0.300 **	−0.264 **	−0.374 **	−0.341 **	0.498 **	0.565 **	-						
11.Age	−0.321 **	−0.160 *	−0.135	−0.143	−0.165 *	−0.131	−0.148 *	0.202 **	0.130	0.121	-					
12.Gender	0.123	0.139	0.183 *	0.078	0.138	0.199 **	−0.020	0.067	−0.136	−0.049	−0.262 **	-				
13.Children	−0.313 **	−0.302 **	−0.268 **	−0.284 **	−0.280 **	−0.259 **	−0.122	0.160 **	0.158 *	0.143	0.435 **	−0.321 **	-			
14. C19_ff	0.201 **	0.146 *	0.185 *	0.072	0.163 *	0.055	0.027	0.065	0.034	0.059	−0.020	0.138	0.006	-		
15. C19_hour per day	0.177 *	0.206 **	0.246 **	0.161 *	0.173 *	0.037	−0.009	0.022	−0.021	0.048	−0.083	−0.092	−0.047	0.093	-	
16. C19_death	0.197 **	0.261 **	0.304 **	0.180 **	0.254 **	0.110	−0.016	0.130	0.091	0.103	−0.078	−0.074	0.001	0.215 **	0.671 **	-

Abbreviations: PSS = Perceived Stress Scale; STSS = Secondary Traumatic Stress Scale; MBI-EE = Maslach Burnout Inventory Health Services Scale, Emotional Exhaustion; MBI-D = Maslach Burnout Inventory Health Services Scale, Depersonalization; MBI-PA = Maslach Burnout Inventory Health Services Scale, Personal Accomplishment; GSE = General Self-Efficacy Scale; RS-14 = 14-item Resilience Scale; C19_ff = Family members or friends infected by COVID19; C19_hour per day = Hour per day with COVID-19 patients; C19_death = Exposed to patients with COVID-19 death. Bold data indicate significant correlations. * *p* < 0.05; ** *p* < 0.01. Bold: highlight significant correlations.

**Table 5:** Questionnaire scales and sub-scales scores in subgroups.

	Gender			Children			Family Members or Friends Infected		
	Mean (SD)			Mean (SD)			Mean (SD)		
	Male	Female	*p*-Value	None	One or More	*p*-Value	None	One or More	*p*-Value
	n = 90	n = 93		n = 53	n = 131		n = 127	n = 57	
PSS	16.02 (6.07)	17.56 (6.43)	0.098	19.19 (6.03)	15.84 (6.13)	**0.001**	16.04 (6.31)	18.51 (5.89)	**0.013**
STSS	34.59 (11.48)	38.15 (13.85)	0.060	42.09 (13.80)	34.11 (11.66)	**<0.001**	35.25 (12.76)	39.00 (12.61)	0.066
Intrusion	9.69 (3.50)	11.18 (4.49)	**0.013**	12.04 (4.68)	9.81 (3.64)	**0.003**	10.01 (4.07)	11.44 (3.97)	**0.028**
Avoidance	14.07 (5.03)	14.89 (5.60)	0.296	16.64 (5.61)	13.63 (4.96)	**<0.001**	14.20 (5.39)	15.16 (5.13)	0.262
Arousal	10.83 (3.96)	12.08 (4.94)	0.062	13.42 (4.92)	10.67 (4.08)	**0.001**	11.04 (4.38)	12.40 (4.66)	**0.057**
MBI-EE	17.43 (9.95)	21.86 (11.85)	**0.007**	23.66 (12.04)	18.05 (10.34)	**0.002**	19.24 (10.51)	20.60 (12.43)	0.447
MBI-D	7.66 (5.06)	7.44 (5.96)	0.793	8.89 (6.44)	6.98 (5.01)	**0.033**	7.46 (5.65)	7.67 (5.22)	0.819
MBI-PA	31.91 (8.76)	33.01 (7.59)	0.365	30.96 (7.88)	33.10 (8.22)	0.108	32.28 (8.76)	32.93 (6.69)	0.621
GSE	31.53 (5.43)	29.98 (5.97)	0.067	29.32 (6.74)	31.33 (5.19)	**0.031**	30.46 (5.95)	31.40 (5.21)	0.302
RS-14	79.20 (11.76)	77.95 (13.64)	0.507	76.45 (14.27)	79.48 (11.97)	0.144	78.06 (12.69)	79.82 (12.79)	0.386
	**Care of COVID-19 Patients**			**COVID-19 Patients’ Death**					
	**Mean (SD)**			**Mean (SD)**					
	**Frontline**	**Other Units**	***p*-Value**	**Yes**	**No**	***p*-Value**			
	**n = 118**	**n = 66**		**n = 94**	**n = 90**				
**PSS**	**17.00 (6.48)**	**16.45 (5.90)**	**0.573**	**17.78 (6.40)**	**15.79 (6.00)**	**0.031**			
STSS	37.61 (13.14)	34.27 (11.95)	0.090	39.72 (12.95)	32.96 (11.73)	**<0.001**			
Intrusion	10.99 (4.14)	9.48 (3.82)	**0.016**	11.51 (4.12)	9.34 (3.76)	**<0.001**			
Avoidance	14.84 (5.35)	13.89 (5.24)	0.249	15.66 (5.38)	13.29 (5.00)	**0.002**			
Arousal	11.78 (4.75)	10.89 (3.98)	0.201	12.55 (4.70)	10.32 (4.01)	**0.001**			
MBI-EE	19.32 (10.75)	20.27 (11.82)	0.579	20.67 (11.17)	18.61 (11.03)	0.210			
MBI-D	6.86 (5.25)	8.71 (5.80)	**0.029**	7.55 (5.76)	7.50 (5.27)	0.948			
MBI-PA	32.99 (8.34)	31.58 (7.81)	0.260	33.33 (7.56)	31.60 (8.70)	0.151			
GSE	31.10 (5.55)	30.12 (6.04)	0.267	31.15 (5.69)	30.33 (5.79)	0.336			
RS-14	79.26 (12.65)	77.44 (12.82)	0.352	79.36 (13.36)	77.82 (12.01)	0.413			

Abbreviations: same abbreviations as Table 4. *p* Values from test T. Bold data indicate differences that are significant.

**Table 6:** Significant Pearson correlation coefficients r: associations between Intrusion and Depersonalization, in F-HCWs vs. OU-HCWs.

	F-HCWs n = 118	OU-HCWs n = 66
	r_s_	
STSS_Intrusion		
PSS	0.559 **	0.503 **
MBI-EE	0.632 **	0.490 **
MBI-D	0.516 **	0.437 **
GSE	−0.279 *	−0.152
RS-14	−0.194 *	−0.363 **
Age	−0.268 **	0.081
Gender	0.263 **	0.119
Children	−0.286 **	−0.284 *
C19_hour per day	0.214 *	0.051
C19_death	0.303 **	0.088
MBI-D		
PSS	0.468 **	0.362 **
STSS	0.520 **	0.501 **
MBI-EE	0.642 **	0.506 **
MBI-PA	−0.253 **	−0.060
GSE	−0.333 **	0.048
RS-14	−0.268 **	−0.442 **
Age	−0.197 *	−0.063
C19_hour per day	0.197 *	0.059

Abbreviations: same abbreviations as Table 4. Bold data indicate correlations that are significant. * *p* < 0.05; ** *p* < 0.01.

**Table 7:** Results of stepwise multiple linear regression analysis predicting secondary traumatic stress (STSS) in the total cohort (n = 184) and Intrusion in F-HCWs (n = 118) vs. OU-HCWs (n = 66).

Dependent Variable	Predictors	β	S.E.	Lim. Inf. (95%)	Lim. Sup. (95%)	*p* Values	Tolerance	VIF
Total cohort								
STSS	Intercept	12.489	1.869	8.800	16.177	<0.001 **		
	PSS	0.754	0.125	0.507	1.000	<0.001 **	0.674	1.485
	MBI-EE	0.489	0.070	0.352	0.626	<0.001 **	0.692	1.445
	C19_death	2.446	0.886	0.698	4.194	0.006 **	0.961	1.040
F-HCWs								
Intrusion	Intercept	3.671	0.822	2.043	5.299	<0.001 **		
	PSS	0.155	0.053	0.050	0.261	0.004 **	0.637	1.569
	MBI-EE	0.118	0.038	0.042	0.193	0.003 **	0.454	2.204
	MBI-D	0.144	0.070	0.005	0.284	0.043 *	0.559	1.790
	Gender	1.322	0.574	0.185	2.460	0.023 *	0.930	1.075
	C19_death	0.821	0.403	0.023	1.619	0.044 *	0.928	1.078
OU-HCWs								
Intrusion	Intercept	3.579	1.186	1.209	5.948	0.004 **		
	PSS	0.257	0.072	0.114	0.400	0.001 **	0.869	1.150
	MBI-D	0.193	0.073	0.047	0.339	0.010 **	0.869	1.150

Note: Variables for STSS: PSS, MBI-EE, MBI-D, Age, Children, C19_ff, C19_hour per day, C19_death, GSE, RS-14. Variables for Intrusion in F-HCWs: PSS, MBI-EE, MBI-D, Age, Gender, Children, C19_hour per day, C19_death, GSE, RS-14. Variables for Intrusion in OU-HCWs: PSS, MBI-EE, MBI-D, RS-14, Children. Abbreviations: same abbreviations as Table 4. β = unstandardized beta, S.E. = standard error, VIF = variance inflation factor. Bold data indicate significant variables in regression model. * *p* < 0.05; ** *p* < 0.01.

## Data Availability

The data presented in this study are available on request from the corresponding author. The data are not publicly available due to privacy issue.
